# Startling stimuli increase maximal motor unit discharge rate and rate of force development in humans

**DOI:** 10.1152/jn.00115.2022

**Published:** 2022-07-13

**Authors:** Jakob Škarabot, Jonathan P. Folland, Aleš Holobar, Stuart N. Baker, Alessandro Del Vecchio

**Affiliations:** ^1^School of Sport, Exercise and Health Sciences, Loughborough University, Loughborough, United Kingdom; ^2^Versus Arthritis Centre for Sport, Exercise and Osteoarthritis, Loughborough University, Loughborough, United Kingdom; ^3^Faculty of Electrical Engineering and Computer Science, University of Maribor, Maribor, Slovenia; ^4^Medical Faculty, Newcastle University, Newcastle upon Tyne, United Kingdom; ^5^Department Artificial Intelligence in Biomedical Engineering, Friedrich-Alexander University, Erlangen-Nürnberg, Erlangen, Germany

**Keywords:** ballistic contractions, EMG decomposition, motoneuron, reticular formation, StartReact

## Abstract

Maximal rate of force development in adult humans is determined by the maximal motor unit discharge rate, however, the origin of the underlying synaptic inputs remains unclear. Here, we tested a hypothesis that the maximal motor unit discharge rate will increase in response to a startling cue, a stimulus that purportedly activates the pontomedullary reticular formation neurons that make mono- and disynaptic connections to motoneurons via fast-conducting axons. Twenty-two men were required to produce isometric knee extensor forces “as fast and as hard” as possible from rest to 75% of maximal voluntary force, in response to visual (VC), visual-auditory (VAC; 80 dB), or visual-startling cue (VSC; 110 dB). Motoneuron activity was estimated via decomposition of high-density surface electromyogram recordings over the vastus lateralis and medialis muscles. Reaction time was significantly shorter in response to VSC compared with VAC and VC. The VSC further elicited faster neuromechanical responses including a greater number of discharges per motor unit per second and greater maximal rate of force development, with no differences between VAC and VC. We provide evidence, for the first time, that the synaptic input to motoneurons increases in response to a startling cue, suggesting a contribution of subcortical pathways to maximal motoneuron output in humans.

**NEW & NOTEWORTHY** Motor unit discharge characteristics are a key determinant of rate of force development in humans, but the neural substrate(s) underpinning such output remains unknown. Using decomposition of high-density electromyogram, we show greater number of discharges per motor unit per second and greater rate of force development after a startling auditory stimulus. These observations suggest a possible subcortical contribution to maximal in vivo motor unit discharge rate in adult humans.

## Graphical abstract

**Figure:**
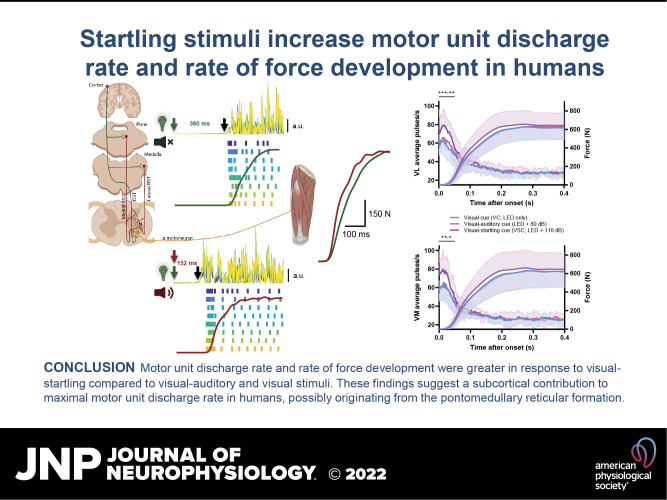

## INTRODUCTION

The rate of muscle force output is predominantly dictated by the central nervous system (1, 2). Unlike in neonates that modulate the rate of force development by higher motor unit synchronization (3), the maximal rate of force development in adult humans is determined by the speed of recruitment and discharge rate of motoneurons (1, 2, 4, 5). Compared with slower, sustained contractions, the motor unit discharge rate during rapid force production is substantially higher and peaks around the onset of force generation (2, 4) followed by a nonlinear decrease similar to in vitro spike frequency adaptation observed in rat (6). Rapid contractions therefore allow insight into the maximal in vivo motoneuron recruitment speed and discharge rate, however the origin of inputs underlying maximal motoneuron discharge rate remains unclear.

**Figure 1.**
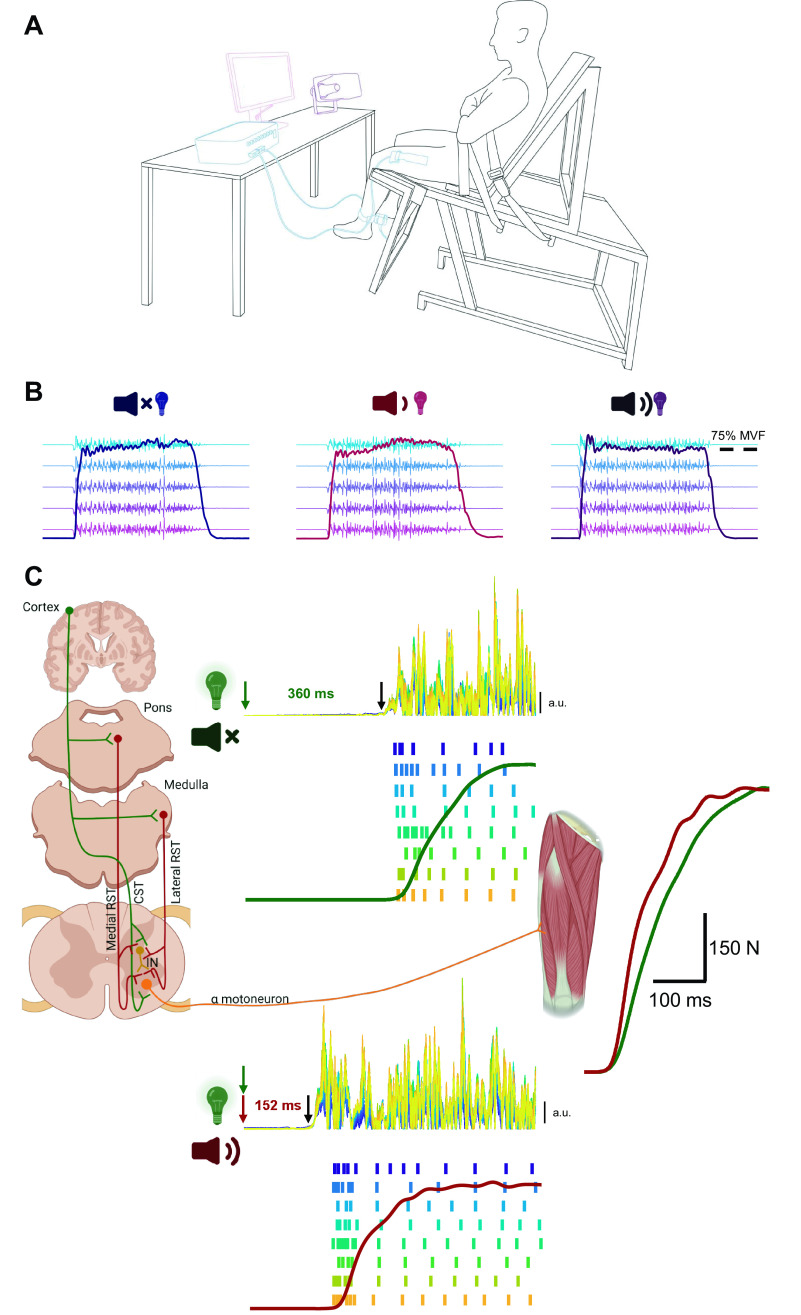
Experimental setup and descending pathways involved in the generation of the motoneuron discharge underpinning maximal rate of force development. *A*: participants were seated in a custom-made isometric dynamometer while high-density electromyography (EMG) recordings were performed on vastus lateralis and medialis. Participants received either a visual (illumination of a light emitting diode, LED), visual-auditory (LED + control acoustic stimulus, 80 dB), or visual-startling (LED + loud acoustic stimulus, 110 dB) cue to which they had to respond by contracting the knee extensors “as fast and as hard” as possible. *B*: examples of force and EMG recordings (recordings from only a single row of the high-density EMG grid placed on vastus lateralis are shown) in response to visual (*left*), visual-auditory (*center*), and visual-startling cue (*right*). *C*: possible descending pathways implicated in producing excitatory synaptic inputs leading to motor unit discharge in the period before force generation. Presentation of a startling auditory stimulus (110 dB; red) purportedly activates neurons in pontomedullary reticular formation that transmit the activation signal to α motoneurons via the reticulospinal tract (RST), drastically shortening the reaction time (the time delay between stimulus delivery and visually determined onset of rectified EMG activity), increasing the initial discharge rate of motoneurons, and resulting in greater rate of force development. CST, corticospinal tract; IN, interneuron. The center panel of *C* shows the rectified EMG amplitude of all channels of high-density EMG (overlaid) and a raster plot of decomposed motor unit pulse trains (each row/color denotes discharges of an individual recruited motor unit) along with the force trace during the performance of rapid contractions in response to visual (*top*) and visual-startling cue (*bottom*). All traces are time-aligned with respect to the timing of visual stimulus. The same force traces are displayed on the right, but in an amplified resolution and time-aligned with respect to the visually determined force onset for better comparison of differences in rate of force development. MVF, maximal voluntary force. Image created with BioRender and published with permission.

Rapid generation of force production is a feedforward task as evidenced by the recruitment of most motoneurons before force onset (2, 4, 5, 7, 8). In the period before force onset, the maximal motoneuron recruitment speed and discharge rate reflect the combination of intrinsic motoneuron excitability (9, 10) and transformation of the excitatory synaptic input without the influence of afferent feedback from the muscle (2, 11). Descending inputs involved in motor command generation with connections to lower motoneurons seem likely substrates of characteristically strong excitatory synaptic input underlying rapid muscle force generation (Fig. 1*C*). These involve cerebral centers, such as pyramidal tract neurons descending from the primary motor cortex. In addition, subcortical neuronal populations such as the pontomedullary reticular formation play an important part in motor command generation (12), the production of gross movement (13–15), and have been implicated in tuning locomotor speed (16). Neurons in the reticular formation are fast-conducting (17), with extensive branching of axons that make mono- and disynaptic connections with motoneurons (18). Reticulospinal neurons are characteristically command neurons (17), and have been shown to be involved in escape movement of vertebrate species where the need for rapid force production is required (19, 20). Both corticospinal and reticular neurons modulate their discharge with contraction strength, however, reticular cells seem to provide an overall level of drive that increases monotonically with force, whereas corticospinal cell discharge is better suited to fine force control (21).

Startling stimuli result in shortening of the reaction time (22) and augmented muscle force output (23, 24). Though the precise mechanisms of these enhancements are as yet unclear, they are thought to represent an involuntary release of preplanned movement stored in subcortical circuits (22, 25–28), likely in the pontomedullary reticular formation (26, 29–31). As such, responses to startling stimuli have been used to infer reticulospinal contribution to human movement. For example, performance of gross motor actions that have at least a moderate reliance on reticulospinal input is accompanied by greater shortening of reaction time in response to startling stimuli compared with fine motor actions that have a greater reliance on corticospinal inputs (26, 32, 33).

In this study, adult humans were required to produce high isometric forces as rapidly as possible in response to presentation of a visual (VC), visual-auditory (VAC), or visual-startling cue (VSC). We tested the hypothesis that the maximal motoneuron output will increase in response to a visual-startling cue, a stimulus that purportedly activates neurons in the pontomedullary reticular formation, which will lead to more rapid generation of muscular force. Using a validated approach to high-density electromyography (HDsEMG) decomposition during rapid contractions (2), we show greater rate of force development, and for the first time, greater number of discharges per motor unit per second in response to a startling auditory stimulus, suggesting subcortical contribution to maximal motoneuron output in humans.

## MATERIALS AND METHODS

### Participants

Twenty-two healthy, recreationally active men (means ± SD; age: 24 ± 2 yr, stature: 1.79 ± 0.07 m, body mass: 80.9 ± 9.5 kg) volunteered to participate in this study. Participants were free from musculoskeletal or neuromuscular injury affecting function of the major joints of the lower limb, were not taking any medication known to affect the nervous system, and reported no hearing-related impairments. The study was approved by Loughborough University Ethical Committee (2021-1749-3524) and was conducted in accordance with the Declaration of Helsinki except for registration in a database. Written, informed consent was provided by participants before taking part in any experimental procedures.

### Experimental Design

Participants visited the laboratory on two occasions where they performed voluntary isometric knee extension contractions with their dominant limb. During the first session, participants were familiarized with the experimental procedures by practicing maximal voluntary and rapid contractions (up to 10 trials and/or until they could reproduce valid contractions three consecutive times; for criteria indicating valid trials, see *Force signal* of *Data Processing and Analysis*), and the StartReact protocol (for details, see the next paragraph). Two to seven days later, participants returned to the laboratory for the experimental session. Participants were instructed to avoid any strenuous activity involving lower limbs within the 48 h before the experimental session and caffeine consumption on the day of testing.

The experimental session started with a warm-up consisting of seven submaximal isometric knee extensions (3 × 50%, 3 × 75%, and 1 × 90% of perceived maximal voluntary force). Following warm-up, participants were instructed to perform a maximal effort isometric knee extension to estimate maximal voluntary force (MVF). Two trials were performed (60 s rest between trials) with strong verbal encouragement and live visual feedback of force level provided. If the two trials differed by more than 5%, an additional trial was performed. The highest instantaneous value was taken as MVF. Following MVF determination, participants performed up to five rapid submaximal practice trials to get used to producing the force as quickly as possible. After that, participants performed the StartReact protocol, adapted from Fisher et al. (34), involving 18 rapid contractions in a block randomized order, with three blocks of six contractions. Participants were instructed to perform rapid contractions in response to illumination of a light emitting diode (LED; 20 ms) placed ∼1.5 m in front of the participant. Out of six rapid contractions in a block, LED was presented either alone (visual cue, VC; two trials), with a control acoustic stimulus (visual-auditory cue, VAC; 80 dB, 500 Hz, 20 ms; RH40V, Adastra, Lisbum, UK; two trials), or a startling acoustic stimulus (visual-startling cue, VSC; 110 dB, 500 Hz, 20 ms; two trials), in a randomized order. Although responses to both visual-auditory and visual-startling cue are likely to be mediated by cochlear nuclei, the visual-startling cue is thought to have an especially powerful effect on the reticular formation (27, 30). A handheld sound level meter was used in pilot testing to ensure consistent and sufficient sound level with respect to the distance of the participant to the sound system, which remained consistent throughout the experiment. Rapid contractions were separated by a random delay of 30–35 s (using customized scripts in Spike2 software; v9, Cambridge Electronics Design Ltd., Cambridge, UK). To avoid decrements in attention, participants were not required to maintain the focus on LED throughout the entirety of a block of contractions; rather they received a “get ready” command from the investigators ∼10 s before LED was due to be illuminated. Before commencing the rapid contractions, participants were presented with five consecutive startling acoustic stimuli (110 dB) every few seconds without performing any contractions for familiarization with the startling cue (34). For each rapid contraction, participants were instructed to contract “as fast and as hard as possible” to the target force of 75% of MVF in response to the “go” signal (illumination of LED) and maintain the force level at 75% MVF target for ∼3 s. The “hold” portion of the task was performed to increase the contraction duration for the purposes of HDsEMG decomposition algorithm that requires identification of sufficient number of independent sources (i.e., motor unit action potentials) from the electromyogram (2, 35). Participants were additionally instructed to maintain a steady resting baseline force avoiding any countermovement or pre-tension (≤0.5 N) before the “go” signal. Visual feedback of force was provided along with magnified feedback of the baseline force level (to facilitate trials without countermovement or pre-tension), and the first derivative of the force signal (as an indicator of the rate of force development achieved) was provided as performance feedback. The experimental set up along with an example of raw force and HDsEMG recordings is shown in Fig. 1, *A*and *B*, respectively. Notably, and despite previous suggestions to the contrary (11), our pilot testing (*n* = 16 men) using the same experimental setup, instructions to the participants, and visual feedback indicated that rapid contractions with a sustained force production of ∼3 s at the target level resulted in similar rates of force development compared with rapid contractions with immediate relaxation upon reaching the target force level (*t* test, *P* ≥ 0.171; 324 ± 100 vs. 321 ± 91, 650 ± 123 vs. 662 ± 118, 388 ± 60 vs. 402 ± 59%MVF·s^−1^ in the 0–50, 50–100, and 100–150 ms time window following force onset, respectively).

### Experimental Procedures

#### Force recordings.

Participants were seated in a rigid custom-made isometric dynamometer designed to measure knee extensor forces. The knee and hip were flexed at 115° and 126°, respectively (180° = full extension). The selected knee angle has been previously shown to maximize isometric knee extension force production (36). Participants were additionally strapped to the dynamometer across the chest and pelvis to prevent extraneous movement. The dynamometer utilized in this experiment has been shown to minimize joint angle changes during maximal isometric efforts (≤4°, compared with >15° of commercial dynamometers; 37). The dominant leg was strapped to a metal brace placed behind the shank at ∼15% of tibial length (lateral malleolus to the knee joint center) above the ankle, and in series with a calibrated S-beam strain gauge (Force Logic, Swallowfield, UK) positioned posterior and perpendicular to the tibia. The analogue force signal was amplified (370 times), sampled at 2,048 Hz, and acquired via a 16-bit multichannel amplifier (Quattrocento; OT Bioelettronica, Torino, Italy). The analogue force signal was also simultaneously sampled with a separate analogue-to-digital converter (2,000 Hz, Micro 1401-3 and Spike2 v10 software, CED Ltd., Cambridge, UK) to display live force feedback to the participant, and to synchronize the recordings for calculation of the reaction time.

#### High-density electromyography.

Multichannel, high-density surface electromyogram was recorded from vastus lateralis (VL) and vastus medialis (VM) muscles. Following skin preparation involving shaving, light abrasion, and cleaning with ethanol, semi-disposable grids of 64 electrodes (13 rows × 5 columns, 1 mm electrode diameter, 8 mm inter-electrode distance; GR08MM1305, OT Bioelettronica, Torino, Italy) were placed over the muscle bellies of vastus medialis and lateralis, with the long axis of the bidimensional grid oriented in line with the orientation of the muscle fibers as described previously (38). The placement of electrode grids on the skin was facilitated by disposable biadhesive foam layers (SpecsMedica, Battipaglia, Italy), the cavities of which were filled with conductive paste (AC Cream, SpecsMedica). A dampened strap ground electrode was placed on the ankle of the nondominant leg, with reference electrodes (Kendall Medi-Trace, Canada) placed over the patella. High-density surface electromyogram signals were bandpass filtered (10–500 Hz), sampled at 2,048 Hz, and recorded in a monopolar configuration via a 16-bit multichannel amplifier (Quattrocento; OT Bioelettronica, Torino, Italy) using OT Biolab+ software (OT Bioelettronica, Torino, Italy).

### Data Processing and Analysis

#### Force signal.

During the offline analysis, the raw voltage signal from the strain gauge was initially converted to force (N), and the baseline of the signal was gravity corrected. The force signal was then filtered with a zero-lag lowpass Butterworth filter with a cut-off frequency of 400 Hz. The onset of force was determined visually by a trained investigator using a systematic method outlined previously (39, 40). Briefly, the signal was displayed with the *x*-axis set to 300 ms before the increase in force from resting levels, and the *y*-axis set to 0–1 N. The force onset was then defined as the first instant of deflection of the force signal away from the envelope of the baseline noise. Such an approach is considered more accurate compared with automated methods (40). Following determination of force onset, the force signal was additionally filtered by a zero-lag third-order Butterworth filter with a cut-off frequency of 20 Hz, to eliminate high-frequency noise but maintain the undistorted force output (41). Trials were considered valid if no countermovement or pre-tension (≤0.5 N) was displayed, and the force level was sufficiently high (≥75% MVF). Out of all trials, the three trials that were both valid and had the highest force at 150 ms after onset per the type of cue (VC, VAC, and VSC) were selected for full analysis. The selection of the three best trials ensured that we captured maximal performance in response to specific cues. For the selected trials, the force signal was analyzed in the first 200 ms following force onset; maximal rate of force development was calculated by the first derivative of force in each overlapping time window ranging from 1 to 200 ms (RFD0-X_max_, N·s^−1^) as described previously (2). Such calculation of the first derivative by varying time windows allows for the assessment of the shifts in the maximal slope of the force-time curve among trials. In addition, force was measured at 50, 100, and 150 ms following force onset, and the rate of force development was calculated as the first derivative of force for fixed time intervals from force onset to 50 ms, 50–100 ms, and 100–150 ms (41, 42), in the interest of comparison with the literature. Because rate of force development has been shown to be related to peak force production achieved during isometric tasks, peak force was also computed for all trials, defined as the greatest instantaneous force produced. All variables were averaged across the three trials per the type of cue.

#### High-density electromyography.

During offline analysis (MATLAB R2021a; MathWorks Inc., MA), monopolar high-density EMG signals were initially bandpass filtered (20–500 Hz) with a fifth-order, zero-lag Butterworth filter. Channels exhibiting poor signal-to-noise ratio, presence of movement artifact, poor skin-electrode contacts, or any irregularities were removed (typically ≤ 5% of all channels) using a semiautomated custom-made tool in MATLAB based on area under the power spectrum and amplitude.

##### Decomposition.

**Figure 2.**
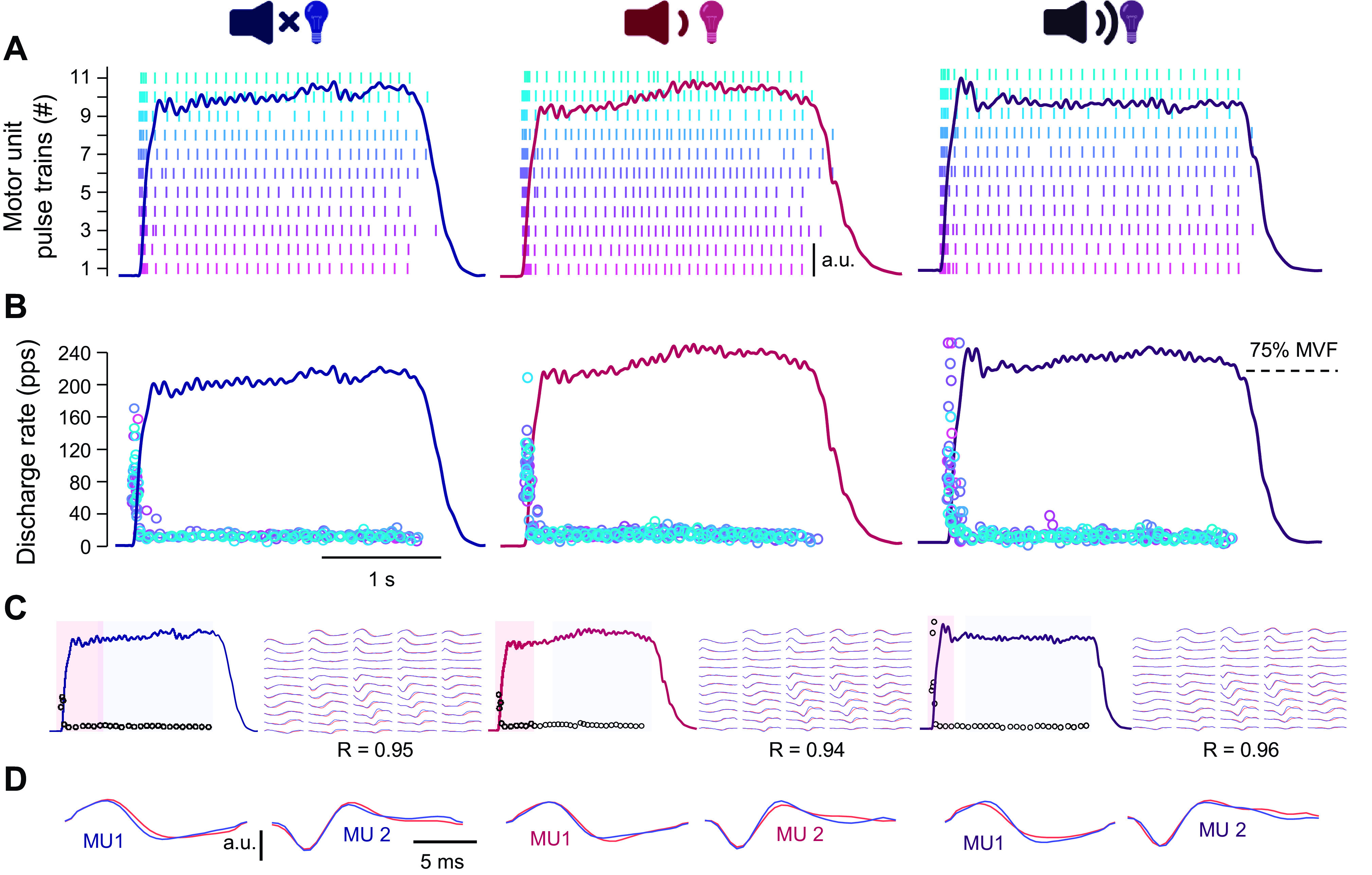
Decomposition of multichannel, high-density electromyogram. High-density surface electromyograms of vastus medialis obtained during rapid contractions in response to visual (LED only; *left*), visual-auditory (LED + 80 dB; *center*), and visual-startling cue (LED + 110 dB; *right*) were decomposed into motor unit pulse trains (*A*), from which the discharge timings could be identified (*B*). To ensure validity of decomposition, spike triggered averaging was performed to compare the first 10 discharges (motor unit discharges denoted by black open circles in the red shaded area; during transient force rise phase) to the final 20 discharges (motor unit discharges denoted by black open circles in the blue shaded area; plateau phase) of individual motor units via two-dimensional cross-correlation (*C*). Examples of motor unit action potentials of two motor units are shown in *D* for comparison of similarity within-contraction (motor unit action potentials overlaid in red and blue as per *C*) and between-contractions (the two motor units displayed in *D* were tracked across conditions and are thus the same across the columns). MVF, maximal voluntary force.

Monopolar HDsEMG signals were decomposed into motor unit pulse trains (Fig. 2*A*) via the Convolution Kernel Compensation algorithm (43). The validity of this algorithm has been repeatedly shown on both synthetic and experimental signals (35, 44). To improve decomposition accuracy, the nine selected contractions (3 for each type of cue) were concatenated before decomposition. Concatenation was performed in a random order, with the order of concatenation stored for viewing only after all the analyses had been completed. The randomization of concatenation ensured the investigator performing editing of motor unit pulse trains (45) was blinded to the experimental conditions, thereby minimizing investigator bias. To further optimize decomposition results, the editing process was performed on three contractions at the time, with motor unit spatial filters acquired during this process applied to the remaining contractions in overlapping windows (46). Only motor units exhibiting a reliable discharge pattern with a pulse-to-noise ratio ≥ 30 dB (accuracy > 90%; false alarm rate < 5%) were retained (35). To further test the validity of the decomposition, the motor unit action potential shapes (obtained via spike-triggered averaging) during the initial (transient force rise phase; first 10 discharges) and last 20 discharges (plateau phase of a contraction) were two-dimensionally cross-correlated (Fig. 2, *C* and *D*; 2, 47). An example of accurate identification of discharge timings following decomposition and further processing from one participant is demonstrated in Fig. 2, *A*–*D*. To further assess the consistency in motor unit pulse identification, we additionally calculated the standard deviation and coefficient of variation of interspike interval of the first five motor unit discharges. Variability of the interspike interval has previously been used as a signal performance metric for decomposition (44, 48), with higher variability typically associated with greater decomposition errors (49). Similarly, we calculated the standard deviation of the delay between the first discharge of the motor unit that was recruited first and the onset of force (i.e., neuromechanical delay) across three contractions per conditions to determine the consistency of the identification of the first motor unit discharge.

From the identified motor units several variables were computed. Maximal discharge rate of motor units was defined as the maximal instantaneous discharge rate at any point during the contraction, though this was very likely to occur within the initial few discharges. Discharge rate at recruitment was defined as the average discharge rate of the first five discharges, indicating discharge rate around force onset, which has been shown to be highly associated with maximal rate of force development (2). Furthermore, we calculated the sum of the discharge timings in a moving 35 ms epoch (corresponding to previously reported delay between onset of motor unit discharge and force), shifted every 1 ms up to 400 ms from the first discharge. The value of the summated discharge timings was then normalized to the time epoch (35 ms) and the number of active motor units, thus constituting the average number of discharges per motor unit per second (2). Finally, we estimated correlation between pulse trains of motor units in the time domain with an approach similar to the one described previously (50). Briefly, we initially computed pulse trains in 100-ms intervals from the first motor unit discharge for 400 ms with 1 ms overlap. The pulse trains were then filtered with a Hann window of 25 ms corresponding to a bandwidth of ∼40 Hz. From there, discrete, progressively increasing groups of filtered motor unit pulse trains were cross correlated across a random set of permutations. To provide estimates of motor unit synchronization, we extracted the averaged cross-correlation value during the first 100 ms of the motor unit pulse trains corresponding to the initial synchronization value, and the averaged cross-correlation of the final 100 ms of the computed time series to estimate synchronization during motor unit discharge at the plateau region of rapid contractions. The analysis of motor unit synchronization was limited to participants with at least a pair of decomposed motor unit discharges (*n* = 14 for VL, *n* = 10 for VM). All variables were averaged across the three trials per type of cue.

##### Residual EMG activity.

**Figure 3.**
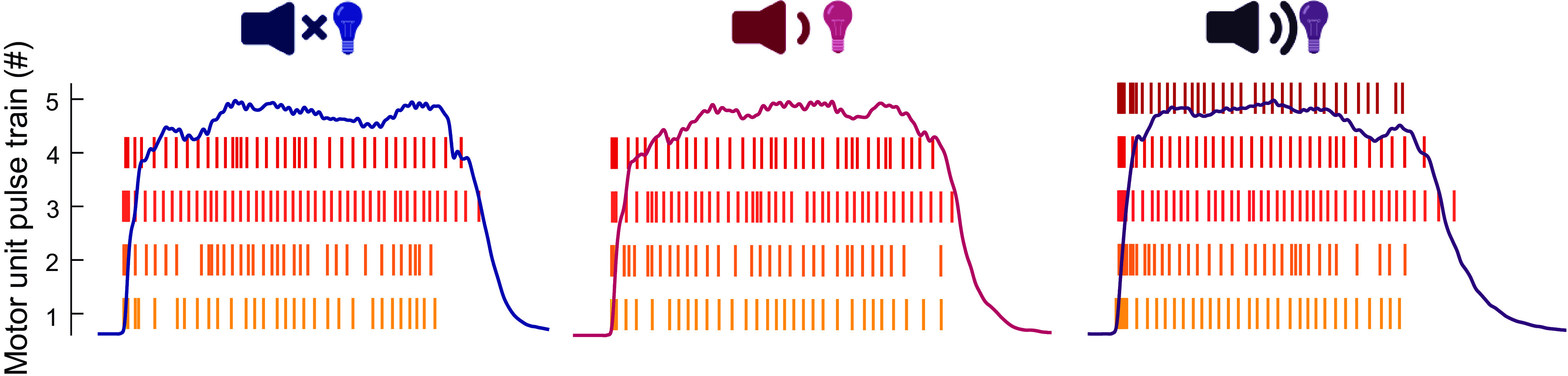
Example individual exhibiting additional motor unit recruitment in response to startling stimuli. An example participant in whom a visual-startling cue resulted in recruitment of an additional motor unit (*right*) in vastus lateralis that was not recruited in response to visual (*left*) and visual-auditory cue (*center*).

Although motoneuron recruitment has been shown to be one of the key determinants of rate of force development, we found only a single individual who exhibited discharges of an additional motor unit exclusively recruited in response to a startling stimulus (Fig. 3). Considering that the current decomposition algorithms only allow discrimination of the activity of a limited proportion of the motor pool, with a bias toward neurons innervating superficial muscle fibers with large potentials (51), it is possible that neurons recruited exclusively in response to startling stimuli that contributed to augmented mechanical outcomes (see results) were undetected.

To check whether additionally recruited units might have been missed by decomposition, analyses of residual monopolar EMG activity were performed as follows. We first estimated motor unit action potentials by calculating spike triggered average of EMG signals, with the identified motor unit discharges used as triggers. After that, the motor unit action potentials of a given channel were convolved with the identified motor unit discharge patterns yielding the motor unit action potential train for each identified motor unit. For each EMG channel, the motor unit action potential trains of all the identified motor units were subtracted from the original EMG signals, yielding the residual EMG activity. This reflects the portion of the EMG signal that was not decomposed into identified unit. The root mean square of the residual EMG activity was then computed in a moving 35 ms epoch from EMG onset, shifted every 1 ms, and averaged across the three trials per type of cue. For the purpose of the statistical analyses, only the values of residual EMG activity in first 50 ms after force onset were compared across conditions as the root mean square EMG amplitude in this time window is most strongly associated with the rate of force development (42).

##### Reaction time and electromechanical delay.

Because stimuli (visual, visual-auditory, or visual-startling) were delivered via a different system to the one with which EMG activity was recorded, the force signal that was simultaneously sampled and recorded in both (via two amplifiers) was initially resampled to the common sampling frequency of 2,048 Hz, then afterward cross-correlated to temporally align the recordings. From there, the reaction time was calculated as the delay between stimulus delivery and visually determined onset of rectified EMG activity of all recorded HDsEMG channels (for example traces, see Fig. 1*C*). The onset of rectified EMG activity was determined with the same systematic procedure used for determination of force onset. To confirm the specificity of the response to a visual-startling stimulus, which, unlike the visual-auditory stimulus, is thought to activate reticulospinal neurons in addition to cochlear nuclei, the gain in motor output in response to the visual-startling cue (VSC gain) was calculated as follows (32):

$$\text{VSC gain}=\frac{\text{VC}-\text{VSC}}{\text{VC}-\text{VAC}},$$

where the numerator is the difference in reaction times in response to visual (VC) and visual-startling cue (VSC) and represents the shortening of the reaction time with the startling stimulus, and the denominator is the difference in reaction times in response to visual-auditory (VAC) and visual cue, which represents the shortening of the reaction time with the auditory stimulus. The electromechanical delay was calculated as the time difference between the onset of force and rectified EMG activity.

### Statistical Analysis

Statistical analyses were performed using SPSS (v27; IBM, IL). Normality of data was assessed with the Shapiro–Wilk test. Residual EMG activity and VSC gain were not normally distributed. A one-sample Wilcoxon signed rank test was therefore performed to assess whether the calculation of the reticulospinal gain was greater than 1. Residual EMG data were transformed with a common logarithm function to meet the assumption of normality. A one-way repeated-measures ANOVA was performed to assess the differences in signal-based decomposition metrics, reaction times, maximal rate of force development, peak force during rapid contractions, the number of discharges per motor unit per second, discharge rate, motor unit synchronization, and residual EMG activity between the three types of cues. A two-way (type of cue: VC, VAC, VSC; time: 0–50, 50–100, 100–150 ms) repeated-measures ANOVA was performed to assess the differences in force and rate of force development in preselected time points/windows. A Greenhouse–Geisser correction was used if the assumption of sphericity (Mauchly’s test) was violated. If significant *F*-values for main effects or interactions were found, analyses were continued with comparisons using least significant difference testing. Significance was set at an α level of 0.05. All data are presented as means ± standard deviation unless stated otherwise.

## RESULTS

### Reaction Time and Electromechanical Delay

**Figure 4.**
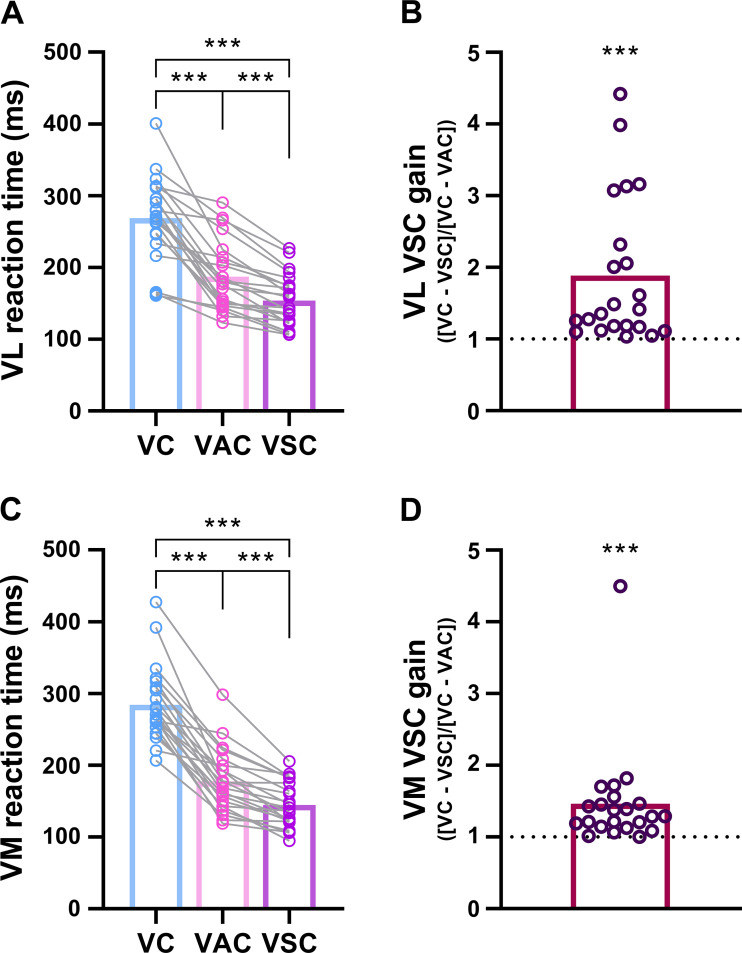
Reaction time and visual-startling cue gain. *Left*: reaction times calculated as the time difference between the delivery of visual (VC; LED only), visual-auditory (VAC; LED + 80 dB), and visual-startling (VSC; LED + 110 dB) cues and the onset of electromyographic (EMG) activity in vastus lateralis (VL; *A*) and vastus medialis (VM; *C*). *n* = 22 men; ***P < 0.001 compared with other types of cues (one-way ANOVA). *Right*: the gain in motor output in response to the visual-startling cue (VSC gain) calculated as the difference in reaction time in response to visual and visual-startling cue relative to the difference in reaction time in response to visual and visual-auditory cue for the VL (*B*) and VM (*D*). *n* = 22 men; ***P < 0.001 compared with 1 (Wilcoxon signed rank test).

Reaction time was influenced by the type of cue in both VL (*F*_1.3,26.5 _= 87.5, *P* < 0.001; Fig. 4*A*) and VM (*F*_1.4,30.0 _= 155.2, *P* < 0.001; Fig. 4*C*), such that the fastest response was after the visual-startling cue (VL: 153.8 ± 36.3 ms; VM: 144.5 ± 31.5 ms) compared with both visual-auditory (VL: 187.0 ± 48.6 ms, *P* < 0.001; VM: 177.6 ± 43.4 ms, *P* < 0.001) and visual cues (VL: 268.8 ± 58.0 ms, *P* < 0.001; VM: 284.0 ± 52.1 ms). Furthermore, the reaction time in response to a visual-auditory cue was shorter than a visual cue (*P* < 0.001 for both VL and VM). The reaction times to the three type of cues are comparable with those reported previously in a quadriceps femoris muscle of healthy adults (52). The gain in motor output in response to the visual-startling cue was significantly greater than 1 in both VL (*P* < 0.001; range 1.04–4.42; Fig. 4*B*) and VM (*P* < 0.001; range 1.00–4.50; Fig. 4*D*). The difference in the onset of force and EMG activity (i.e., the electromechanical delay) did not differ across conditions for either the VL (20.2 ± 8.3 vs. 21.1 ± 5.8 vs. 22.8 ± 6.8 ms in response to visual, visual-auditory, and visual-startling cue, respectively; *F*_2,42 _= 1.4, *P* = 0.268) or VM (26.0 ± 9.2 vs. 28.3 ± 10.2 vs. 29.2 ± 17.3 ms; *F*_1.5,31.7_ = 0.9, *P* = 0.382).

### Mechanical Output

**Figure 5.**
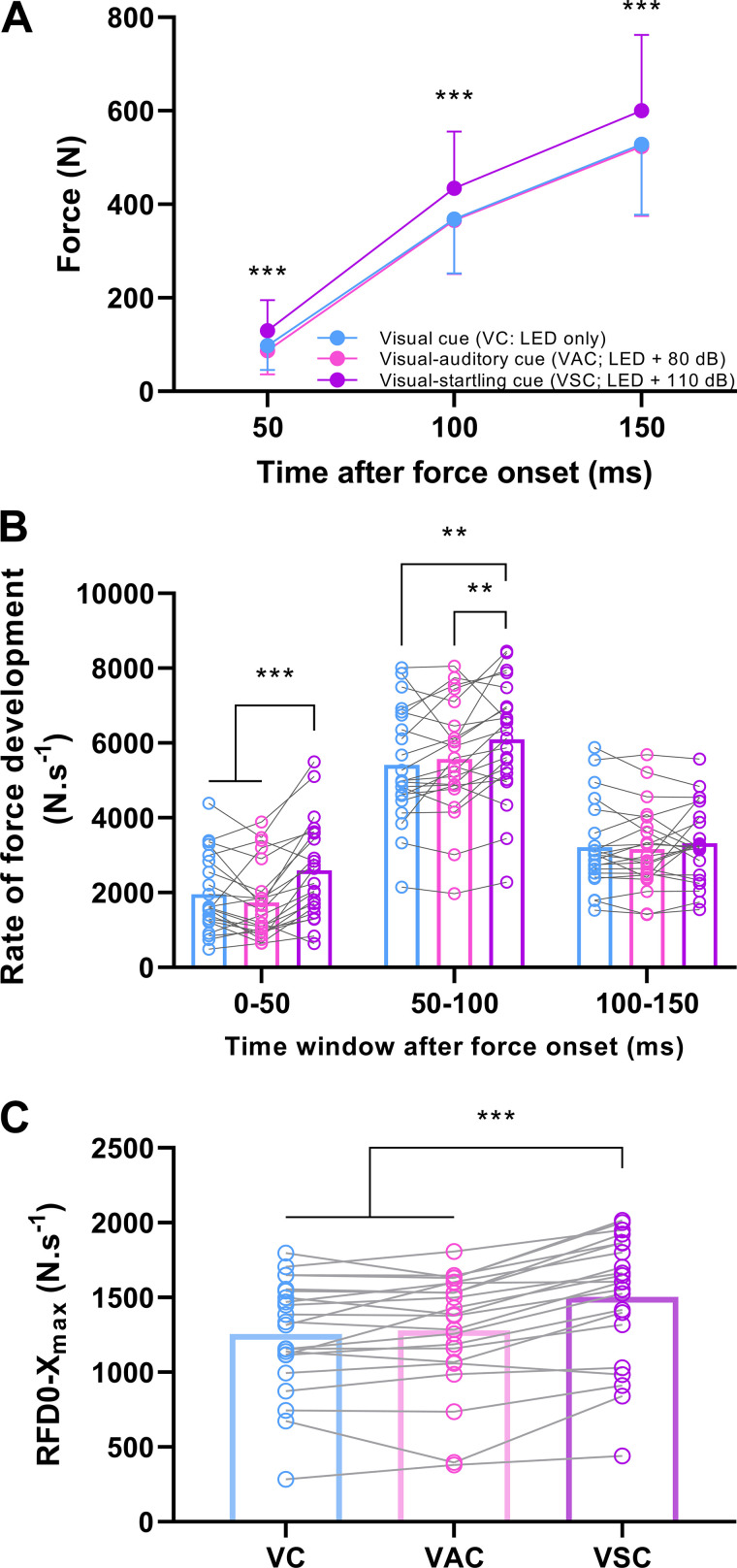
Force and rate of force development. Force produced at fixed time points following force onset (*A*), rate of force development (RFD; *B*) during fixed time windows following force onset (individual responses with means), and maximal rate of force development (*C*) achieved during the entire force-time curve (individual responses with means in bars) in response to either a visual cue (VC; LED only), visual-auditory cue (VAC; LED + 80 dB), or visual-startling cue (VSC; LED + 110 dB). *n* = 22 men; ***P < 0.001, **P < 0.010 for visual-startling relative to other types of cues (one-way ANOVA).

There was an effect of the type of cue for absolute force at fixed time points following force onset (*F*_2,42 _= 29.5, *P* < 0.001), rate of force development during fixed time windows following force onset (*F*_2,42 _= 28.4, *P* < 0.001), and maximal rate of force development achieved during the entire force-time curve (*F*_2,42 _= 48.4, *P* < 0.001). Specifically, force production was greater at 50, 100, and 150 ms following force onset, respectively, when responding to the auditory-startling cue compared with visual and visual-auditory cue (*P* < 0.001 for all; Fig. 5*A*), with no difference between the latter two type of cues at any time point (*P* ≥ 0.530). Similarly, rate of force development was greater in response to the auditory-startling cue compared with the other two types of cues in the 0–50 ms (both *P* < 0.001) and 50–100 ms (*P* ≤ 0.006) time window following force onset (Fig. 5*B*), with no differences detected in the rate of force development between the visual and visual-auditory time cues during the same time windows (*P* ≥ 0.153). There were no differences in rate of force development when responding to different types of cues in the 100–150 ms time window (all *P* ≥ 0.279). Maximal rate of force development was greater in response to the auditory-startling cue compared with visual and visual-auditory cues (both *P* < 0.001; Fig. 5*C*), with no differences in maximal rate of force development between the latter cue types (*P* ≥ 0.358). The peak force level was similar in response to visual (500.9 ± 146.0 N), visual-auditory (506.5 ± 148.5 N), and visual-startling cues (509.1 ± 145.7 N; *F*_1.5, 31.1 _= 0.9, *P* = 0.365).

### Motor Unit Decomposition

Decomposition yielded reliable motor unit identification in 17 and 14 participants (out of a total sample of 22) in VL and VM, respectively. A total of 68 motor units were identified in the VL with the analysis yielding an average of 4.0 ± 2.2 motor units per contraction per individual (range 1–9). The average two-dimensional coefficient of correlation, indicating similarity of VL motor unit action potential shapes between the transient phase of force rise and the plateau region of the contraction, was 0.87 ± 0.07, with no differences detected during contractions in response to different types of cues (*F*_1.4,22.4 _= 2.1, *P* = 0.154). There were no differences across experimental conditions in standard deviation (26.7 ± 8.8, 27.2 ± 15.1, and 27.3 ± 8.4 ms in response to visual, visual-auditory, and visual-startling cue, respectively; *F*_1.3,22.6 _= 0.1, *P* = 0.903) and coefficient of variation (0.38 ± 0.14, 0.39% ± 0.16%, and 0.41 ± 0.12%; *F*_2,32 _= 1.5, *P* = 0.232) of interspike intervals of the first five VL motor unit discharges, suggesting consistency in motor unit pulse identification. There was also no bias identified across experimental conditions when considering the standard deviation of the delay between the first discharge of the VL motor unit that was recruited first and the onset of force production (i.e., the SD of neuromechanical delay; 7.4 ± 5.0 vs. 7.5 ± 4.9 vs. 7.5 ± 5.6 ms; *F*_2,32 _= 0.002, *P* = 0.998).

Within the VM, a total of 70 motor units were identified, with an average of 5.0 ± 3.8 motor units per respective contraction per individual (range 1–11). The average two-dimensional correlation coefficient in the VM was 0.90 ± 0.08 and was not different during contractions in response to different types of cues (*F*_2,26 _= 1.2, *P* = 0.323). No differences were found across conditions regarding standard deviation (35.8 ± 15.8, 35.2 ± 13.5, and 33.2 ± 12.0 ms; *F*_1.3,16.8 _= 0.5, *P* = 0.522) and coefficient of variation (0.46 ± 0.16%, 0.44 ± 0.13%, and 0.47 ± 0.13%; F_2,26 _= 0.8, *P* = 0.471) of interspike intervals of the first five VM motor unit discharges. The standard deviation of the neuromechanical delay also did not differ across experimental conditions in VM (8.7 ± 7.4 vs. 9.8 ± 8.7 vs. 8.8 ± 9.0 ms; F_1.2, 15.3 _= 0.1, *P* = 0.758), suggesting consistency in identifying the first motor unit discharge.

### Motor Unit Discharge Modulation

**Figure 6.**
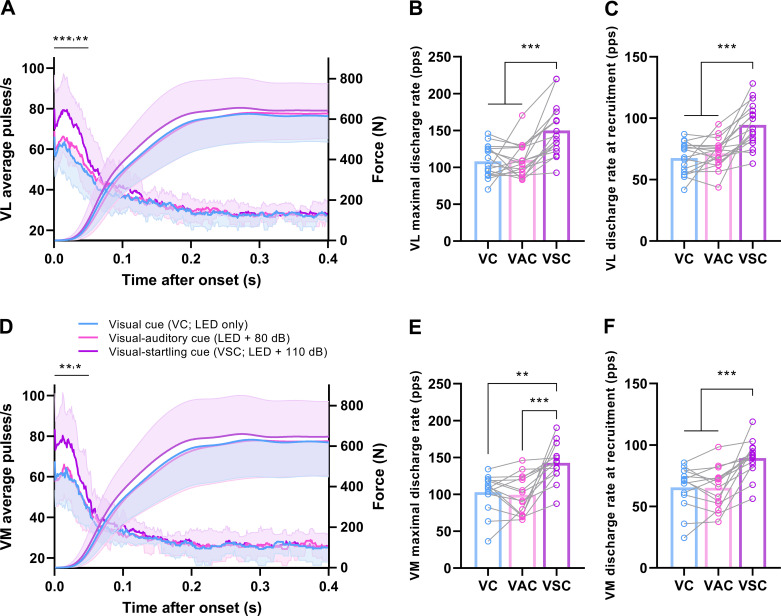
Motor unit discharge rate and force. The average number of discharges per motor unit per second, measured within a 35-ms moving window from the first discharge to 400 ms, in vastus lateralis (VL, *n* = 17 men; *A*) and vastus medialis (VM, *n* = 14 men; *D*), and the force output in response to a visual (LED only), visual-auditory (LED + 80 dB) and visual-startling (LED + 110 dB) cue from force onset. Note that on the *x*-axis average pulses/s are plotted from the first motor unit discharge, whereas force is plotted from force onset. Shaded area represents standard deviation. Note the significant difference in response to the visual-startling cue relative to the other two types of cues in the first 50 ms, a time period corresponding to the greatest differences in rate of force development. Individual responses with means (bar plots) of the maximal instantaneous discharge rate (VL, *B*; VM, *E*) and the discharge rate at recruitment (mean of first five discharges; VL, *C*; VM, *F*) during rapid contractions in response to visual, visual-auditory, and visual-startling cue are also displayed. ***P < 0.001, **P < 0.010, *P < 0.05 for visual-startling relative to other types of cues (one-way ANOVA).

The average number of discharges per motor unit per second, with respect to mechanical output is shown in Fig. 6. The average value of the number of discharges per motor unit per second in the 50 ms from the first discharge was affected by the type of cue in VL (*F*_2,32 _= 16.4, *P* < 0.001; Fig. 6*A*) and VM (*F*_1.3,17.2_ = 10.5, *P* = 0.002; Fig. 6*D*), with it being greater in response to visual-startling cue compared with visual-auditory (VL: *P* = 0.002; VM: *P* = 0.020), and visual cue (VL: *P* < 0.001; VM: *P* = 0.007), consistent with the time window of the largest differences in mechanical output between cues (i.e., RFD within the first 50 ms after force onset). No differences in the number of discharges per motor unit per second were detected between visual-auditory and visual cue (VL: *P* = 0.346; VM: *P* = 0.889).

The maximal discharge rate was affected by the type of cue in both the VL (*F*_2,32 _= 18.4, *P* < 0.001; Fig. 6*B*) and VM (*F*_2,26 _= 14.9, *P* < 0.001; Fig. 6*E*), and was greater in response to visual-startling compared with visual-auditory (*P* < 0.001 for both VL and VM) and visual cue (VL: *P* < 0.001; VM: *P* = 0.001), but no differences were detected between responses to the visual-auditory and visual cues (VL: *P* = 0.797; VM: *P* = 0.593). Similarly, discharge rate at recruitment, constituting the mean discharge rate during the first five discharges, differed in response to different cues in both VL (*F*_2,32 _= 23.7, *P* < 0.001; Fig. 6*C*) and VM (*F*_2,26 _= 18.0, *P* < 0.001; Fig. 6*F*). Specifically, the discharge rate at recruitment was greater in response to visual-startling compared with visual-auditory and visual cue (*P* < 0.001 for all). There were no differences detected in the discharge rate of the first five discharges between visual-auditory and visual cue (VL: *P* = 0.274; VM: *P* = 0.884). The discharge rate at the plateau phase of the contraction did not differ across conditions in both VL (17.3 ± 1.6 pps; *F*_2,32 _= 0.1, *P* = 0.917) and VM (17.5 ± 2.8 pps; *F*_2,26 _= 1.8, *P* = 0.196).

**Figure 7.**
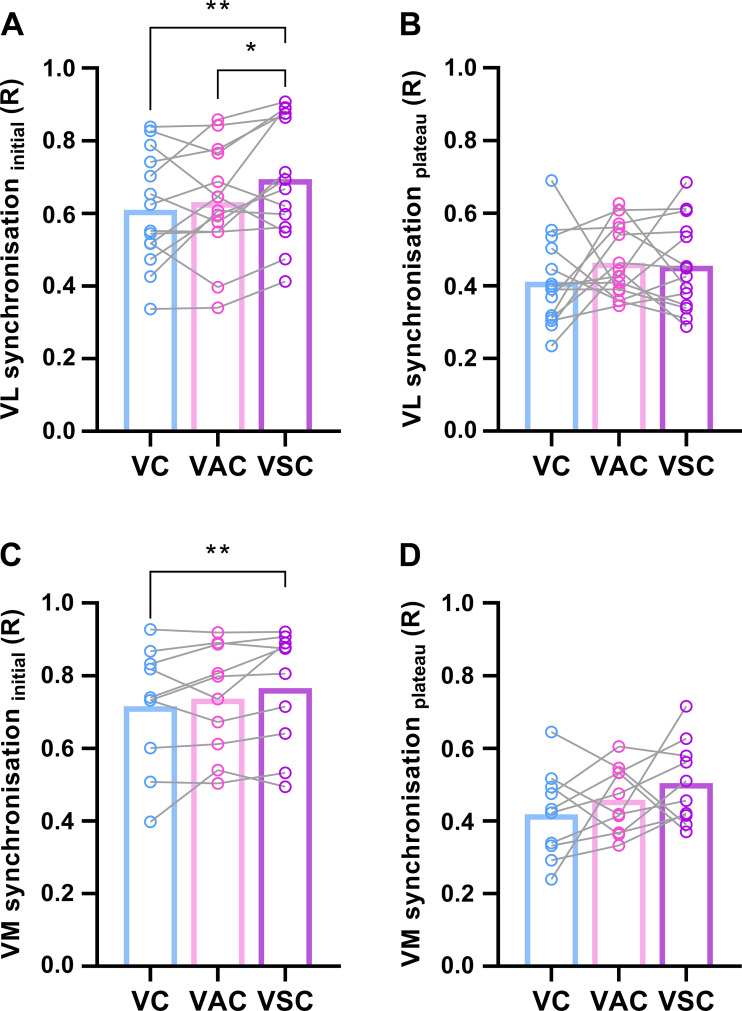
Motor unit synchronization. Motor unit synchronization in vastus lateralis (VL; *n* = 14 men; *A* and *B*) and medialis (VM, *n* = 10 men; *A* and *B*) during the performance of rapid contractions in response to visual (VC), visual-auditory (VAC), and visual-startling cue (VSC). Synchronization was calculated for the first 100 ms of motor unit discharge, corresponding to the transient increase in force (Synchronization initial; *A* and *C*), and for 100 ms during a plateau phase of rapid contractions (Synchronization plateau; *B* and *D*). **P < 0.010, *P < 0.05 relative to other cues (one-way ANOVA).

Motor unit synchronization during the transient force increase was affected by the type of cue in both VL (*F*_2,26 _= 6.5, *P* = 0.005; Fig. 7*A*) and VM (*F*_2,20 _= 3.9, *P* = 0.038; Fig. 7*C*). In VL, motor unit synchronization was greater in response to visual-startling compared with visual-auditory (*P* = 0.021) and visual cues (*P* = 0.003), with no differences between the latter two cues (*P* = 0.451). In VM, motor unit synchronization was greater in response to visual-startling compared with visual cue (*P* = 0.010), but not visual-auditory cue (*P* = 0.122), with no differences between visual-auditory and visual cue (*P* = 0.339) The type of cue did not affect motor unit synchronization during the plateau phase of rapid contractions in both VL (*F*_2,26 _= 1.0, *P* = 0.368; Fig. 7*B*) and VM (*F*_2,18 _= 1.8, *P* = 0.198; Fig. 7*D*).

### Residual EMG Activity

**Figure 8.**
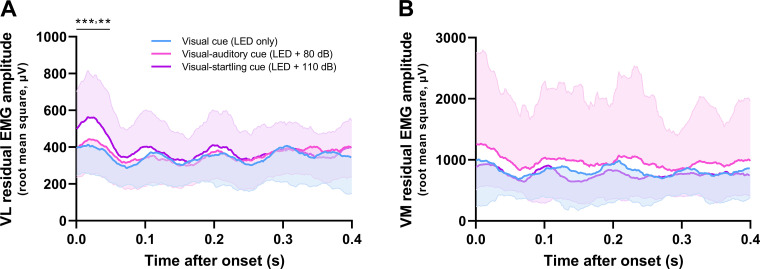
Residual electromyographic (EMG) activity. Residual EMG was computed by subtracting motor unit action potential trains (obtained from the decomposition) from the original EMG signal. Root mean square amplitude of the residual EMG signal was then calculated with a moving 35-ms window from EMG onset for 400 ms in response to visual (LED only), visual-auditory (LED + 80 dB), and visual-startling cue (LED + 110 dB) for both vastus lateralis (VL; *A*) and medialis (VM; *B*). Statistical analyses were performed in the first 50 ms following onset due to the association of the EMG activity in this time window with the rate of force development. *n* = 17 men for VL, *n* = 14 men for VM. ***P < 0.001, **P < 0.010 relative to other types of cues (one-way ANOVA).

Residual EMG activity was affected by the type of cue in the VL in the first 50 ms from EMG onset (*F*_2,32 _= 8.6, *P* = 0.001; Fig. 8*A*). Post hoc testing showed that the root mean square value of residual EMG signal was greater in response to the visual-startling compared with both the visual-auditory (*P* = 0.005) and visual cues (*P* < 0.001), with no differences between visual-auditory and visual cues (*P* = 0.902). However, there was no effect of cue on the residual EMG activity of the VM (*F*_1.5,23.3 _= 0.2, *P* = 0.756; Fig. 8*B*). This analysis suggests that some additional units were likely recruited in response to the visual-startling cue, at least in VL, but these were not successfully decomposed by the algorithm.

## DISCUSSION

In this study, we tested a novel hypothesis that the maximal motor unit discharge rate will increase in response to a startling cue, a stimulus that purportedly activates neurons in the pontomedullary reticular formation. We demonstrated that the presentation of a visual-startling cue shortened the reaction time and increased the number of discharges per motor unit per second and the rate of force development. The increased motor unit discharge rate in response to a visual-startling cue suggests a possible subcortical contribution to maximal in vivo motor unit discharge rate in humans.

### Startling Stimuli Increase Motor Unit Discharge and Rate of Force Development

A higher number of discharges per motor unit per second and greater rate of force development were only evident when responses to the visual-startling cue were compared with the visual and visual-auditory cues, with no differences between the latter two types of cues. Given that responses to a visual-auditory cue will be mediated by the cochlear nuclei and could thus result in intersensory facilitation (53), the observation that augmented motor unit discharge rate and mechanical output were specific to the visual-startling stimulus further supports the notion that mediation occurred via increased descending input to motoneurons. Notably, the differences in neural and mechanical outputs were consistently the largest in the first 50 ms following respective onsets, which has been previously shown to be associated with descending neural mechanisms (42).

Startling stimuli have been shown to augment force output and increase the rate of force development (23). However, the rate of force development and motor unit discharge rate are related to the peak force achieved during the contraction (4, 10), thus the greater rate of force production reported previously (23) might be an artifact of greater absolute force. In the present study, the peak force levels did not differ across all conditions, providing convincing evidence that the augmentation of the neural drive and the rate at which muscle force can be produced is a true function of a startling stimulus.

The discharge rate of motor units during rapid contractions was the highest around recruitment, with a large range of maximal instantaneous discharge rates between individuals as shown previously (2). In the present study, we extended these observations by demonstrating that the motor unit discharge rate can be significantly enhanced when a startling stimulus is presented. The greater motor unit discharge rate in response to visual-startling stimuli suggests that the neural substrate augmented by startling stimuli is responsible for high discharge rate that typically underpins force production during rapid tasks. Given that startling stimuli purportedly activate neurons in the pontomedullary reticular formation (26, 29, 30), the increase in motor unit discharge rate might reflect transformation of inputs transmitted via the reticulospinal pathway that makes both monosynaptic and disynaptic connections to motoneurons (18).

Motor unit recruitment has previously been shown to be a key factor in maximal rate of force development (1, 2). However, we found only a single individual who exhibited discharges of an additional motor unit exclusively recruited in response to a startling stimulus (Fig. 3), making it possible that higher-threshold units that contributed to augmented mechanical outputs in response to startling stimuli were largely undetected by decomposition. Such a hypothesis is consistent with the greater residual EMG amplitude in response to startling stimuli, at least in the vastus lateralis. Although this greater residual EMG amplitude might reflect a greater discharge rate of undetected units, global surface EMG amplitude has been shown to be more sensitive to changes in motor unit recruitment rather than discharge rate (54). Our analysis of residual EMG amplitude therefore suggests recruitment of higher-threshold motor units during the performance of a rapid, high-force contraction in response to a startling stimulus.

It has been speculated previously that rate of force development could be augmented as a result of increased synchronization of motor unit discharge rate (55). We showed that synchronization of motor unit discharges was greater in response to visual-startling cue during the transient increase in force output of rapid contractions. However, it should be noted that such behavior is more likely to be due to the intrinsic link between greater motor unit discharge rate and synchronization (50, 56). Indeed, we generally found that startling stimuli had greater and more consistent effects on motor unit discharge rate than synchronization, suggesting that the latter is the consequence of the former. Overall, our results are consistent with the notion presented previously that an increase in the rate of force development, facilitated by the startling stimulus in the present study, is a consequence of faster recruitment and greater discharge rate of motor units (1, 2, 4, 5), which might be, at least in part, driven by subcortical structures.

### The Possible Role of Reticulospinal Input in Generating Maximal in Vivo Motoneuron Discharge

The enhanced voluntary response when cued by a startling stimulus in humans differs from a startle reflex in that it does not habituate and does not exhibit prepulse inhibition (57). Randomization of the order of cue presentation, along with highly consistent mechanical and neurophysiological outcomes in the present study make habituation and a learning effect an unlikely mechanism to explain our results. Several studies have suggested that the origin of an augmented response to a startling stimulus is subcortical, likely through the activation of neurons in the pontomedullary reticular formation (26, 29, 30), and we provide indirect support of this supposition via significant and specific shortening of the reaction time in response to a startling stimulus (32).

Both healthy individuals after an intervention targeting the reticular formation (58), and patient populations with extensive cortical damage (59–61) have shown an enhanced StartReact response. Nevertheless, the contribution of cortical influences to startling stimuli cannot be fully excluded (62), and similarly, the performance of a rapid, high-force task likely has a cortical component. For example, Baudry and Duchateau (63) observed a specific response in the preparatory phase of rapid, compared with slower ramp contractions, including a steeper and delayed (i.e., closer to force onset) rise in corticospinal excitability, intracortical disinhibition, with only a limited increase in spinal excitability, suggesting a role of the primary motor cortex in encoding rapid contractions. It should be noted, however, that our results do not necessarily conflict with those of Baudry and Duchateau (63), but rather complement them. Indeed, there is an extensive network of cortico-reticular connections (Fig. 1*C*; 64–66), thus in response to a startling stimulus, cortical inputs will likely be amplified by the reticulospinal neurons leading to a greater motoneuron output. Collectively, the present data, in conjunction with previous work, suggest that different neural substrates, both cortical and subcortical, likely act synergistically and contribute to fast recruitment and high discharge rate of motoneurons as well as their variability during the performance of rapid, high-force contractions.

### Further Considerations and Implications of the Findings

Visual-auditory and visual-startling stimuli shortened the reaction time as shown previously, however, it was only the latter that also resulted in greater rate of force development. These findings suggest a level of decoupling in the programming of human reaction time and maximal rate of force production. Indeed, while reaction times may be influenced by intersensory facilitation (53), the key determinants of the maximal rate of force development are the speed of motor unit recruitment and discharge characteristics in the period before force onset where the influence of afferent feedback is likely to be absent (1, 2). Future studies should consider investigating motor programming strategies in conjunction with startling stimuli between reaction time and the rate of force production using an experimental design that could conceivably decouple the two outcome variables.

In addition to the likely involvement of ionotropic inputs (i.e., those originating from pyramidal tract and reticular formation neurons), neuromodulatory inputs, which modulate the responsiveness of motoneurons to ionotropic inputs (67), might also play a role in the motor unit discharge rate underpinning maximal rate of force development (68). Lesion and pharmacological studies in rats have previously shown noradrenaline to have an excitatory effect on responses to startling stimuli (69–71). However, this effect is less likely in the present study due to slow actions of noradrenaline (72), and the interleaved nature of startling and nonstartling trials in a randomized order that make noradrenaline accumulation from startling stimuli less likely.

The present study provides evidence for the possible subcortical contribution to motor unit discharge characteristics underpinning rapid force production. These findings have potential implications for sports performance as well as populations exhibiting motor impairment. However, it remains unknown if the use of startling stimuli as an intervention strategy leads to improvements in voluntary recruitment and discharge rates of motor units and this should be investigated in the future, with longitudinal studies and consideration for populations with cortical and/or subcortical impairments. Similarly, aging adults exhibit alterations in motor unit morphology (73) and activity (74), and there is some evidence of reticulospinal alterations (75), which might contribute to the age-related decline in rapid force production. Thus, it remains to be seen whether the effect observed in the present study is similar in aging adults, particularly considering presbycusis that might render startling stimuli a less effective stimulus in aging adults.

### Methodological Considerations

There are some methodological limitations in the present study that need to be considered. First, decomposition of multichannel EMG signals during rapid contractions performed at high levels of force output is difficult due to superimposition of many independent sources (i.e., motor unit action potentials), particularly during the transient increase in muscle force whereby erroneous decomposition and selection of false positive motor unit pulses could influence the results of the present study. To validate decomposition results and minimize the potential of erroneous identification of motor unit discharges, we relied on a number of different signal-based metrics. Namely, we assessed the similarity of the motor unit action potential shapes between the transient increase and steady portion of the force output, which has been previously shown to allow for high level of discrimination among motor unit action potentials (47), and we found no differences in action potential shapes within contractions and across conditions. We further assessed consistency in motor unit pulse identification by calculating the variability in the interspike intervals of the first five motor unit discharges, as well as variability of identification of the first discharge of the motor unit that was recruited first, and again found no bias across conditions. When further considering these signal-based metrics with a blinded approach to motor unit editing, it makes it unlikely that any potential erroneous motor unit pulse identification significantly influenced our findings. Nevertheless, it bears noting that decomposition of multichannel EMG signals during rapid contractions remains to be validated by comparison with intramuscular EMG signals.

Second, the decomposition of multichannel EMG signals yielded ∼5 motor units per participant per contraction on average. Although this number of motor units is on average greater than the number typically obtained from intramuscular recordings, it is still substantially lower than the number of units within the whole motor pool (76). Nevertheless, the consistency in the motor unit behavior shown in the present study, namely the consistent increases in discharge rate in response to the visual-startling cue, makes it likely that the decomposed units are relatively representative of the behavior of the motor pool.

Finally, it is important to consider the generalizability of the findings. Although the strength of the reticulospinal inputs has been suggested to vary across muscle groups (77, 78), subsequent studies have shown a wide distribution of reticulospinal inputs (18, 79). Indeed, the StartReact effect has been observed in a variety of muscle groups (26, 32, 52, 58, 80). Therefore, we would expect the effect observed in the present study to be replicated across several muscles and muscle groups, though the magnitude of the effect might vary depending on the muscle.

### Conclusion

We demonstrated that when performing a rapid, high-force isometric task, presentation of a loud (startling) acoustic stimulus increased the average number of motor unit discharges per second and the rate of force production. Though cortical influences cannot be excluded, the increased motor unit discharge rate in response to a visual-startling cue suggests a subcortical contribution to maximal motoneuron output in humans, possibly originating from the pontomedullary reticular formation.

## GRANTS

Dr. J. Škarabot is supported by Versus Arthritis Foundation Fellowship (Ref: 22569). Prof. A. Holobar is supported by Slovenian Research Agency under Grants J2-1731, L7-9421, and P2-0041. Prof. S. Baker is supported by the UK BBSRC under Grant BB/V00896/X1.

## DISCLOSURES

No conflicts of interest, financial or otherwise, are declared by the authors.

## AUTHOR CONTRIBUTIONS

J.Š., J.P.F., A.H., S.N.B., and A.D.V. conceived and designed research; J.Š. performed experiments; J.Š., A.H., and A.D.V. analyzed data; J.Š., J.P.F., S.N.B., and A.D.V. interpreted results of experiments; J.Š. prepared figures; J.Š. drafted manuscript; J.Š., J.P.F., A.H., S.N.B., and A.D.V. edited and revised manuscript; J.Š., J.P.F., A.H., S.N.B., and A.D.V. approved final version of manuscript.
